# Supplementary material for: Finite Element Analysis of a New Non-Engaging Abutment System for Three-Unit Implant-Supported Fixed Dental Prostheses
Source: Bioengineering (Basel). 2022 Sep 20;9(10):483. doi: 10.3390/bioengineering9100483 (PMC9598935; doi:10.3390/bioengineering9100483)
Supplement: Supplementary file 1 [file bioengineering-09-00483-s001.zip › bioengineering-1902181-supplementary.pdf]

Supplement Table S1. Peak von Mises stress and maximal principal stress

Group	Loading	Peak von Mises stress		
		Fixture	Screw	Abutment
AO	Axial	176.122	239.319	506.539
	15°	180.422	256.307	530.299
	45°	194.145	287.772	608.725
BD	Axial	363.056	197.931	158.198
	15°	373.138	194.323	135.517
	45°	472.376	171.256	260.959
**Group**	**Loading**	**Maximal principal stress**		
		Cortical bone	Cancellous bone	Crown
AO	Axial	43.278	6.794	56.102
	15°	50.864	5.568	81.325
	45°	28.698	1.809	28.962
BD	Axial	20.263	2.046	38.683
	15°	17.235	2.319	47.815
	45°	17.628	1.254	25.603

Supplement Table S2. Result data by nodes

Group	Loading		Abutment		Fixture		Cancellous		Cortical	
			2nd M	2nd P	2nd M	2nd P	2nd M	2nd P	2nd M	2nd P
AO	Axial	AVG	21.95	21.23	22.43	24.18	12.15	12.23	113.30	133.57
		SEM	0.212	0.152	0.189	0.183	0.216	0.228	3.582	3.411
	15°	AVG	21.48	23.31	22.60	23.46	13.19	11.40	125.69	130.79
		SEM	0.199	0.245	0.228	0.287	0.236	0.206	4.264	3.201
	45°	AVG	27.55	29.03	21.53	20.30	9.09	8.59	129.64	104.53
		SEM	0.309	0.335	0.375	0.372	0.130	0.103	3.477	2.276
BD	Axial	AVG	33.52	37.10	20.15	20.19	9.78	9.87	86.59	123.82
		SEM	0.779	0.805	0.431	0.456	0.192	0.194	3.276	5.889
	15°	AVG	28.94	30.61	19.33	18.10	10.52	8.83	90.90	114.37
		SEM	0.708	0.706	0.421	0.441	0.206	0.167	3.880	5.265
	45°	AVG	43.96	50.01	32.38	29.21	9.57	8.56	123.24	104.23
		SEM	1.738	1.884	0.994	1.009	0.142	0.120	4.234	3.807

AVG: average, SEM: standard error of the mean, M: molar, P: premolar.
